# Erector spinae plane block reduces postoperative nausea and vomiting in liver surgery

**DOI:** 10.1097/JS9.0000000000001707

**Published:** 2024-05-29

**Authors:** Kuo-Chuan Hung, Li-Chen Chang, I-Wen Chen

**Affiliations:** a Department of Anesthesiology, Chi Mei Medical Center, Tainan City; b Department of Anesthesiology, Chi Mei Medical Center, Liouying, Tainan City; c Department of Anesthesiology, E-Da Hospital, I-Shou University, Kaohsiung City, Taiwan

*Dear Editor,*

We read with interest the article ‘Ultrasound-guided erector spinae plane block for postoperative analgesia in patients after liver surgery: A systematic review and meta-analysis on randomized comparative studies’ by Bhushan *et al*.^1^. This meta-analysis aimed to compare the analgesic efficacy and feasibility of the erector spinae plane block (ESPB) with non-block care or other blocks in patients undergoing liver surgery^1^. The authors found that, based on the analysis of six randomized controlled trials (RCTs) with 392 patients, ESPB did not significantly reduce postoperative pain scores at rest at 8, 24, 48, or 72 h compared to control groups. ESPB also did not significantly decrease postoperative 24 h opioid consumption or improve sleep quality on the first night. However, ESPB reduced the incidence of postoperative nausea and vomiting (PONV)^1^. ESPB is a relatively new block that is technically simple to perform and has a good safety profile; therefore, determining its efficacy compared to established techniques is of clinical interest.

Although this meta-analysis^1^ suggests that ESPB may not provide meaningfully superior analgesia to other approaches in this setting, the finding that ESPB significantly reduced the incidence of PONV compared to the control groups (OR 0.29; *P*=0.001) is clinically important. PONV is a common and distressing complication after surgery that can delay recovery, prolong hospital stay, and decrease patient satisfaction. Identifying techniques such as ESPB that can help prevent PONV is valuable for improving postoperative outcomes. However, with only six RCTs and 392 patients included in this meta-analysis^1^, it is uncertain if there is sufficient evidence to definitively conclude that ESPB should be routinely used specifically for PONV prophylaxis. The limited number of studies and small sample size raises the possibility that the observed benefit could be a spurious result.

Trial sequential analysis (TSA) can be a useful tool to help clarify whether the evidence is conclusive enough to inform clinical practice^2,3^. TSA is a statistical method used to reduce the risk of random errors in cumulative meta-analyses due to repeated significance testing as new data emerges^4^. TSA involves constructing a monitoring boundary, similar to an interim analysis in a clinical trial, which helps determine whether a trial should be terminated early, owing to sufficient evidence being found for a treatment effect^4^. If the cumulative *Z*-curve crosses the TSA monitoring boundary or the required information size boundary, the desired intervention effect is considered statistically significant and potentially conclusive, potentially negating the need for further trials^4^.

To address this issue, we performed TSA using raw data from the original meta-analysis, with settings of *α* 5%, power 80%, and an observed relative risk reduction of 57% for PONV with ESPB vs. control. The TSA results (Fig. 1) showed that the cumulative *Z*-curve crossed the required information size boundary, indicating that sufficient evidence has accrued to conclusively determine that the 57% risk reduction is real, and no further studies are needed. This strengthens the confidence in the meta-analysis findings.

**Figure 1:**
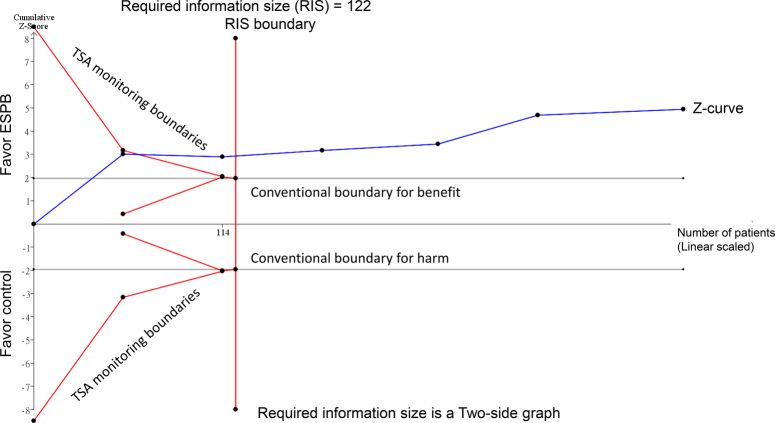
Trial sequential analysis (TSA) assessing the effect of erector spinae plane block (ESPB) vs. control on postoperative nausea and vomiting (PONV) incidence in patients undergoing liver surgery. The required information size of 122 patients was calculated based on a relative risk reduction of 57% in the ESPB group, an *α* of 5%, and a power of 80%. The cumulative *Z*-curve crosses the required information size boundary, indicating that sufficient evidence exists to conclude that ESPB provides a 57% reduction in PONV compared with the control.

In conclusion, the original meta-analysis^1^ found that ESPB significantly decreased the incidence of PONV compared with control treatments in patients undergoing liver surgery. Although the number of studies was limited, TSA supported the idea that sufficient evidence had been accumulated to draw firm conclusions. These results suggest that ESPB may be a useful technique to include in a multimodal approach to PONV prophylaxis for liver surgery patients.

## Ethical approval

Not applicable.

## Consent

Not applicable.

## Source of funding

No external funding was received for this study.

## Author contribution

I-W.C. and K.-C.H.: wrote the main manuscript text; L.-C.C.: prepared Figure 1. All authors read and approved the final version of the manuscript.

## Conflicts of interest disclosure

The authors declare no conflicts of interest.

## Research registration unique identifying number (UIN)

Not applicable.

## Guarantor

Kuo-Chuan Hung.

## Data availability statement

The datasets used and/or analyzed in the current study are available from the corresponding author upon reasonable request.

## Provenance and peer review

This paper was not invited.
